# Pharmacological Profile of Nociceptin/Orphanin FQ Receptors Interacting with G-Proteins and β-Arrestins 2

**DOI:** 10.1371/journal.pone.0132865

**Published:** 2015-08-06

**Authors:** D. Malfacini, C. Ambrosio, M. C. Gro’, M. Sbraccia, C. Trapella, R. Guerrini, M. Bonora, P. Pinton, T. Costa, G. Calo’

**Affiliations:** 1 Department of Medical Sciences, Section of Pharmacology and National Institute of Neuroscience, University of Ferrara, Ferrara, Italy; 2 Department of Pharmacology, Istituto Superiore di Sanità, Rome, Italy; 3 Department of Chemical and Pharmaceutical Sciences and LTTA, University of Ferrara, Ferrara, Italy; 4 Department of Morphology, Surgery, and Experimental Medicine, Section of Pathology, Oncology, and Experimental Biology and LTTA, University of Ferrara, Ferrara, Italy; University of North Dakota, UNITED STATES

## Abstract

Nociceptin/orphanin FQ (N/OFQ) controls several biological functions by selectively activating an opioid like receptor named N/OFQ peptide receptor (NOP). Biased agonism is emerging as an important and therapeutically relevant pharmacological concept in the field of G protein coupled receptors including opioids. To evaluate the relevance of this phenomenon in the NOP receptor, we used a bioluminescence resonance energy transfer technology to measure the interactions of the NOP receptor with either G proteins or β-arrestin 2 in the absence and in presence of increasing concentration of ligands. A large panel of receptor ligands was investigated by comparing their ability to promote or block NOP/G protein and NOP/arrestin interactions. In this study we report a systematic analysis of the functional selectivity of NOP receptor ligands. NOP/G protein interactions (investigated in cell membranes) allowed a precise estimation of both ligand potency and efficacy yielding data highly consistent with the known pharmacological profile of this receptor. The same panel of ligands displayed marked differences in the ability to promote NOP/β-arrestin 2 interactions (evaluated in whole cells). In particular, full agonists displayed a general lower potency and for some ligands an inverted rank order of potency was noted. Most partial agonists behaved as pure competitive antagonists of receptor/arrestin interaction. Antagonists displayed similar values of potency for NOP/Gβ_1_ or NOP/β-arrestin 2 interaction. Using N/OFQ as reference ligand we computed the bias factors of NOP ligands and a number of agonists with greater efficacy at G protein coupling were identified.

## Introduction

Nociceptin/Orphanin FQ (N/OFQ) is a neuropeptide of 17 amino-acids (FGGFTGARKSARKLANQ) that binds with high affinity the N/OFQ peptide (NOP) receptor [1,2]. NOP is a G-protein coupled receptor (GPCR) whose activation leads to the inhibition of both cAMP levels and calcium channels, and to the stimulation of potassium currents; these cellular effects are due to the activation of pertussis toxin (PTX)-sensitive G-proteins (G_i/o_) [3]. NOP receptor shares high degree of structural similarities with opioid receptors. Furthermore, N/OFQ sequence is similar to that of dynorphin A, an endogenous opioid peptide. Despite such similarities, N/OFQ does not bind opioid receptors and NOP does not interact with opioid neuropeptides. Thus, the NOP receptor was defined as opioid-related rather than opioid [4]. Recently, the crystal structure of the human NOP receptor was solved in complex with the antagonist compound-24 (C-24) [5] revealing some substantial differences in the binding pockets of NOP and classical opioid receptors [5,6]. The N/OFQ-NOP system has been deeply investigated by academic and industrial researchers leading to the discovery of a variety of selective NOP receptor ligands [7,8]. Using such compounds the role of this system in physiology and pathology has been, at least partially, elucidated. N/OFQ and the NOP receptor are involved in the regulation of different biological functions at both central and peripheral levels including pain, mood and anxiety, food intake, learning and memory, locomotion, cough and micturition reflexes, cardiovascular homeostasis, intestinal motility and immune responses [3].

It is known that GPCRs may signal not only by interacting with G-proteins but also with other effectors, including arrestins [9]. It is also known that some GPCR ligands may act as biased agonists i.e. ligands that after binding to a single receptor are able to activate with different efficacy some of its pathways over others; for example G-protein over arrestin or vice versa. Thus, based on their ability to dissect the biological actions that follow the activation of a given receptor, biased agonists may act as more effective/better tolerated drugs. For instance, TRV120027 is a AT-1 receptor ligand that behaves as β-arrestin 2 biased agonist. It has been demonstrated that TRV120027, similar to the standard AT-1 antagonists, inhibits angiotensin II-mediated vasoconstriction while, via β-arrestin coupling, it increases cardiomyocyte contractility. This profile of action may provide important benefit in acute heart failure. This proposal has been corroborated by preclinical [10] as well as clinical [11] evidence. More examples of GPCR, biological systems, and possible therapeutic indications for which biased ligands may be valuable are reviewed in [12]As far as the opioid receptor field is concerned, the ability to interact with arrestins has been demonstrated for classical opioid receptors [13] and more recently for the NOP receptor [14]. The biological role of arrestins within the classical opioid receptor field was demonstrated in vivo using mice lacking the β-arrestin 2 gene. Compared to their wild type littermates, these animals displayed a remarkable enhancement and prolongation of the analgesic effect of morphine [15] and a reduced tolerance liability [16]. Based on these findings it has been proposed that opioid receptor biased agonists able to promote receptor/G-protein better than receptor/arrestin interaction may display higher efficacy and/or better tolerability [17]. Of note, TRV130 a recently discovered [18] mu opioid receptor agonist biased towards G-protein displayed a potent analgesic action in animals associated with lower gastrointestinal and respiratory side effects than morphine [19]. This promising profile has been very recently confirmed in patients [20].

Recently we used a novel assay based on the bioluminescence resonance energy transfer (BRET) between Renilla Luciferase (RLuc) and Renilla Green Fluorescent Protein (RGFP) [21] for investigating opioid receptor pharmacology. In particular RLuc was linked to the mu or delta opioid receptors while RGFP to signal transducer proteins (G-protein or arrestin). After testing several peptide and non-peptide opioid ligands marked differences of efficacy for G-protein and arrestin were detected, with a pattern suggesting more restrictive structural requirements for arrestin efficacy [22]. In the present study, we present the first systematic investigation of the pharmacological profile of the NOP receptor interacting with both G-protein and β-arrestin 2. To this aim a large panel of NOP ligands encompassing full (N/OFQ, N/OFQ(1–13)-NH_2_ [23], UFP-112 [24], [Arg^14^Lys^15^]N/OFQ [25], PWT2-N/OFQ [26], Ro 65–6570 [27], SCH 221510 [28]) and partial ([Phe^1^ψ(CH_2_-NH)Gly^2^]N/OFQ(1–13)-NH_2_ ([F/G]N/OFQ(1–13)-NH_2_) [29], UFP-113 [30], Ac-RYYRIK-NH_2_ [31]) agonist as well as pure antagonist ([Nphe^1^]N/OFQ(1–13)-NH_2_ [32], UFP-101 [33], J-113397 [34], SB-612111 [35], C-24 [36]) activity has been evaluated using the above mentioned BRET assay. The results of this investigation allow us to begin the analysis of functional selectivity and biased agonism in the NOP receptor field.

## Materials and Methods

### Drugs

The peptides N/OFQ, N/OFQ(1–13)-NH_2_, UFP-113, UFP-112, UFP-101, [F/G]N/OFQ(1–13)-NH_2_, [Nphe^1^]N/OFQ(1–13)-NH_2_, [Arg^14^Lys^15^]N/OFQ, Ac-RYYRIK-NH_2_, and PWT2-N/OFQ were synthesized in house following the procedures previously described in detail [23,37]. The non-peptide molecules Ro 65–6570, SCH-221510, C-24, and J-113397 were synthesized in our laboratories by Dr Claudio Trapella. Compounds SB-612111, GDP, and naloxone were from Tocris bioscience (Bristol, UK). Pertussis toxin was from List biological laboratories Inc. (Campbell, CA 95008, US). All tissues culture media and supplements were from Invitrogen (Paisley, UK). Reagents used were from Sigma Chemical Co. (Poole, UK) or E. Merck (Darmstadt, Germany) and were of the highest purity available. Native coelenterazine (CLZN, 5 mM, EtOH) was from Synchem UG & Co. KG (Altenburg, Germany). Concentrated solutions of ligands were made in ultrapure water (1 mM, peptides; 10 mM, GDP and naloxone) or dimethyl sulfoxide (10 mM) and kept at - 20°C until use.

### Plasmids

Human NOP Rluc-tagged fusion proteins were made by replacing stop codons with a sequence encoding a 10-mer linker peptide (GPGIPPARAT) and cloned into pRluc-N1 (PerkinElmer, Waltham, MA, USA). NOP-Rluc inserts were then transferred into the retroviral expression vector pQIXN (Clontech, Los Baños, Philippines). Bovine Gβ_1_ N-terminal-tagged with RGFP (Prolume, Pinetop, USA) was built by linking the RGFP sequence without its stop codon to Ser^2^ of Gβ_1_ through a 21-mer linker peptide (EEQKLISEEDLGILDGGSGSG) and cloned into the retroviral expression vector pQIXH. The N terminus of human β-arrestin 2 after removal of the start codon was tethered to the C terminus of RGFP through a 13-mer linker peptide (EEQKLISEEDLRT) and sub-cloned in pQIXH [22].

### Cell and membrane preparation

Human Embryonic Kidney (HEK293) cells were grown in Dulbecco’s modified Eagle’s medium, supplemented with 10% (v/v) fetal calf serum, 100 units/ml penicillin G, and 100 ng/ml streptomycin sulfate, in a humidified atmosphere of 5% CO_2_ at 37°C. Cell lines permanently co-expressing the different pairs of fusion proteins, i.e. NOP-RLuc/Gβ_1_-RGFP and NOP-RLuc/β-arrestin 2-RGFP, were prepared using the pantropic retroviral expression system by Clontech as described previously [21]. For G-protein experiments enriched plasma membrane aliquots from transfected cells were prepared by differential centrifugation; cells were detached with PBS / EDTA solution (1 mM, pH 7.4 NaOH) then, after 5 min 500 g centrifugation, Dounce-homogenized (30 strokes) in cold homogenization buffer (TRIS 5 mM, EGTA 1 mM, DTT 1 mM, pH 7.4 HCl) in presence of sucrose (0.32 M). Three following centrifugations were performed at 1000 g (4°C) and supernatants kept. Two 25,000 g (4°C) subsequent centrifugations (the second in the absence of sucrose) were performed for separating enriched membranes that after discarding the supernatant were kept in ultrapure water at -80°C [38]. The protein concentration in membranes was determined using the QPRO—BCA kit (Cyanagen Srl, Bologna, IT) and the spectrophotometer Beckman DU 520 (Brea, CA, USA).

### Compound interaction with luciferase activity

For assessing whether compounds affect luciferase activity all the ligands were assayed at 1 and 10 μM employing cell membranes obtained from HEK293 expressing the human NOP-RLuc and β-arrestin 2-RGFP. [5 μM of coelenterazine were added together with membranes 15 min before readings and compounds 5 min before readings]. Data were expressed as mean CPS values in 4 readings (~ 60 s delayed) using the 460(25) filter with the microplate luminometer Victor 2030 (PerkinElmer, Waltham, MA, USA).

### Receptor levels

The levels of NOP fusion proteins expressed in transfected cells were determined by measuring RLuc luminescence activity. Dilutions of cell membranes (0.1–4 μg) made in duplicate were counted in the Victor 2030 (PerkinElmer, Waltham, MA, USA) luminometer to detect RLuc emission; 5 μM coelenterazine was automatically injected to each sample, and, after a delay of 2 s, total light emission was counted at 0.5 s intervals for 5 s. Integrated photon counts were plotted as a function of membrane protein concentration and the linear regression of the data has been analyzed.

### Receptor-transducer interaction

In whole cells luminescence was recorded in 96-well sterile poly-D-lysine-coated white opaque microplates, while in membranes it was recorded in 96-well untreated white opaque microplates (PerkinElmer, Waltham, MA, USA). For the determination of NOP/β-arrestin 2 interactions, cells co-expressing NOP-Rluc and β-arrestin 2-RGFP were plated 24 h before the experiment (100,000 cells / well). The cells were prepared for the experiment substituting the medium with Dulbecco’s phosphate buffered saline (DPBS) with 0.5 mM MgCl_2_ and 0.9 mM CaCl_2_. For the determination of NOP/G-protein interaction, membranes (3 μg of protein) prepared from cells co-expressing NOP-Rluc and Gβ_1_-RGFP were added to wells in DPBS. Coelenterazine at a final concentration of 5 μM was always injected 10 minutes prior reading the cell plate. The receptor / G-protein interaction was measured in cell membranes to exclude the involvement of other cellular processes (i.e. arrestin recruitment, internalization). Next, different concentrations of ligands in 20 μL of PBS—BSA 0.01% (Bovine Serum Albumin, Sigma Chemical Co. (Poole, UK)) were added and incubated for an additional 5 min before reading luminescence. In pilot experiments the effects of N/OFQ, UFP-112, and Ro 65–6570 were measured after 5, 10, and 15 min of incubation. Signals were collected using a Victor 2030 luminometer (PerkinElmer, Waltham, MA, USA), emissions were selected using a 460(25) and a 510(10) bandpass filters for Rluc and RGFP, respectively. All the experiments were performed at room temperature. All the experiments were performed at room temperature.

### Assessment of antagonist potency

Compounds that do not display agonist activity were further evaluated as antagonists. Three types of experiments were performed i) concentration-response curves to N/OFQ in absence and in presence of a fixed concentration of antagonist, ii) concentration-response curves to N/OFQ in absence and in presence of increasing concentrations of SB-612111 (Schild analysis), iii) inhibition-response curves to SB-612111 against a fixed concentration of N/OFQ approximately corresponding to its EC_80_.

In pilot experiments performed in cell membranes, 15 min pre-incubation with SB-612111 100 nM, C-24 10 nM, and UFP-101 1 μM were challenged against N/OFQ by measuring BRET ratio 5 min after agonist injection. Concentration response curves to N/OFQ were rightward shifted in the presence of all antagonists, the agonist maximal effect in presence of UFP-101 was not significantly different than control while in the presence of SB-612111 or C-24 the agonist maximal effect was strongly depressed. These experiments were then repeated by increasing to 15 min the time between agonist injection and the measure of BRET ratio. Under these experimental conditions all antagonists produced a rightward shift of the concentration response curve to N/OFQ without modifying agonist maximal effect. Therefore this protocol was adopted for all subsequent antagonist experiments.

### Data analysis and terminology

All data are computed as stimulated BRET ratio units, i.e. the ratio between CPS from RGFP and RLuc in the presence of ligands, followed by baseline subtraction, i.e. the BRET value in the absence of ligand. Agonist potencies are given as pEC_50_ i.e. the negative logarithm to base 10 of the molar concentration of an agonist that produces 50% of the maximal effect of that agonist. Maximal agonist effects (E_max_) were expressed as fraction of the N/OFQ E_max_ which was determined in every assay plate and reported in the graphs as E / E_max_.

Concentration-response curves to agonists and inhibition response curves to antagonists were analyzed with a four-parameter logistic nonlinear regression model:

$$\text{Effect = Baseline + }{\text{(E}}_{\text{max}}\;–\text{ Baseline) / }{\text{(1+10\textasciicircum{}((LogEC}}_{\text{50 }}\;–\text{ Log[ligand]) Slope))}$$

Curve fitting was performed using PRISM 5.0 (Graph Pad Software In., San Diego, USA).

The antagonist potency for SB-612111 in inhibition response experiments was expressed as pK_B_, which was calculated as the negative logarithm to base 10 of the K_B_ from the following equation:

K_B_ = IC_50_ / ([2 + ([A] / EC_50_)^n^]^1/n^– 1), where IC_50_ is the concentration of antagonist that produces 50% inhibition of the agonist response, [A] is the concentration of agonist, EC_50_ is the concentration of agonist producing a 50% maximal response and n is the slope coefficient of the concentration-response curve to the agonist [39].

When antagonists were assayed at a single concentration against the concentration-response curve to the agonist their pK_B_ was derived with the following equation: pK_B_ = log(CR—1)—log[A] where CR is the ratio between agonist potency (expressed as EC_50_) in the presence and absence of antagonist and [A] is the molar concentration of antagonist.

The type of antagonism exerted by SB-612111 was assayed by using the Schild analysis. The Schild plot was analyzed by linear regression to derive the pA_2_ value of the antagonist.

To quantify the differences of agonist efficacies for G protein and arrestin interactions the Bias factors were calculated by choosing the endogenous NOP ligand N/OFQ as standard unbiased ligand. For this analysis, the E_max_ and EC_50_ of the agonist were derived using a 3-parameters logistic model with unitary slope values. In fact, although several agonist curves displayed slope values different from 1, on refitting the curves with the parameter fixed to unity did not produce a statistically significant reduction of the goodness of fit (extra-sum of squares principle [40]). Under such conditions, the relative ratio (E_max_/EC_50_)_lig_ / (E_max_/EC_50_)_N/OFQ_ is equivalent to the relative (τ/K)_lig_/(τ/K)_N/OFQ_ ratio as defined by the operational model [41,42], and represents the ratio of both intrinsic efficacy (i.e., ε as defined in [43]) and binding affinity of the ligands with respect to the reference agonist [44,45]. By taking ratios of these values between G protein and arrestin can cancel the common K and yield the ratio of ligand intrinsic efficacy across the two transduction proteins. Thus, the following formula was used for calculating agonist bias factors in log_10_ units:

$$\text{bias factor }=\text{ log}{\left[{\left({\text{E}}_{\text{max}}/{\text{EC}}_{50}\right)}_{\text{lig}}\;/\;{\left({\text{E}}_{\text{max}}/{\text{EC}}_{50}\right)}_{\text{N/OFQ}}\right]}_{\text{G-protein}}\;-\text{ log}{\left[{\left({\text{E}}_{\text{max}}/{\text{EC}}_{50}\right)}_{\text{lig}}\;/\;{\left({\text{E}}_{\text{max}}/{\text{EC}}_{50}\right)}_{\text{N/OFQ}}\right]}_{\text{β-arr}}$$

Data are expressed as mean ± sem of n experiments and were analyzed statistically using one-way analysis of variance followed by Dunnett’s test for multiple comparisons. Potency values are expressed as mean ± CL_95%_. Bias factors were analyzed statistically using the Student t test for paired data. p values < 0.05 were considered statistically significant.

## Results

### Ligands effect on luciferase activity

NOP receptor ligands used in this study were tested for their effects over RLuc activity in cell membranes. At 1 μM the compounds did not modify RLuc activity. Similar results were obtained by testing the compounds at 10 μM with the only exception of Ro 65–6570 and PWT2-N/OFQ that produced a significant decrease in photons emitted by RLuc. Thus 1 μM was chosen as the highest concentration tested in concentration-response curves to Ro 65–6570 and PWT2-N/OFQ (Table 1).

**Table 1:** Evaluation of CPS emitted in NOP/RLuc expressing membranes in presence of 1 or 10 μM of following compounds.

	1 μM	10 μM
PBS (and BSA 0.01%)	11330 ± 125	12022 ± 692
PBS/DMSO (0.1–0.01%)	11244 ± 756	11381± 137
N/OFQ	10482 ± 675	12315 ± 1833
N/OFQ(1–13)-NH_2_	10662 ± 863	12767 ± 1737
[Arg^14^Lys^15^]N/OFQ	9864 ± 456	11949 ± 1911
UFP-112	13152 ± 436	13995 ± 843
PWT2-N/OFQ	11406 ± 912	*6018 ± 543
[F/G]N/OFQ(1–13)-NH_2_	12950 ± 322	13886 ± 936
UFP-113	12496 ± 659	13455 ± 959
Ac-RYYRIK-NH_2_	12746 ± 989	13555 ± 809
[Nphe^1^]N/OFQ(1–13)-NH_2_	12844 ± 434	13410 ± 566
UFP-101	12432 ± 851	11920 ± 512
Ro 65–6570	12190 ± 356	*7792 ± 563
SCH-221510	11616 ± 787	12012 ± 396
J-113397	10810 ± 663	11791 ± 981
SB-612111	10128 ± 846	11268 ± 1140
C-24	12298 ± 366	12709 ± 411
Naloxone	12146 ±1065	12591 ± 445
GDP	11554 ± 1124	12601 ± 1012

Data are mean ± sem of 3 separate experiments.

* p < 0.05 vs. PBS according to one way ANOVA followed by the Dunnett’s test for multiple comparisons.

### Expression levels of RLuc/NOP receptor in Gβ_1_/RGFP and β-arrestin 2/RGFP expressing cells

**Fig 1:**
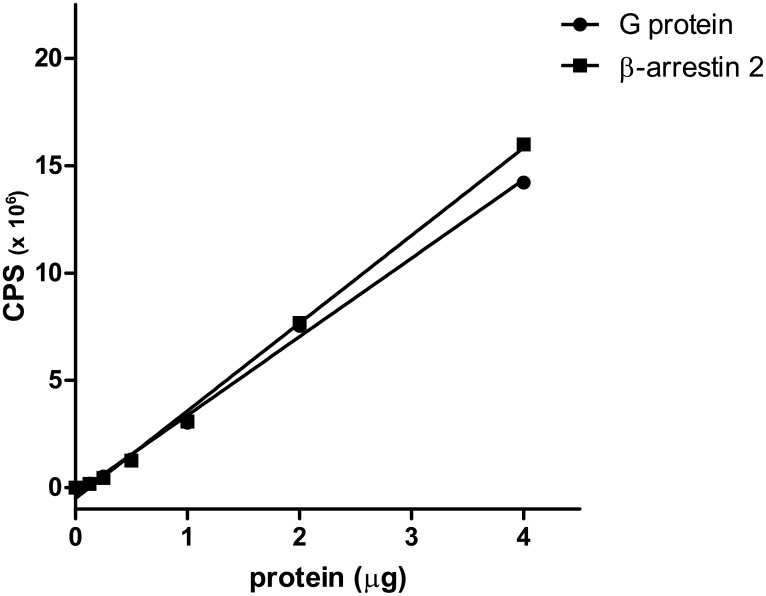
Light emitted (CPS) as function of the amount of protein (μg) in membranes prepared from NOP/RLuc cells expressing either Gβ_1_/RGFP or β-arrestin 2/RGFP membranes.

In order to compare the expression levels of NOP receptor in the two cell lines we measured photons emitted by RLuc in response to 5 μM coelenterazine as a function of membrane protein concentrations. As shown in Fig 1 in both cell lines there was a linear increase in CPS with the increase of the membrane protein concentration. The slopes of the regression lines, 3.66 ± 0.065·10^6^ and 4.09 ± 0.068·10^6^ CPS·10^6^·μg^-1^ for Gβ1 and β-arrestin 2 cells, respectively, were not significantly different (Fig 1).

### Assessment of NOP/G-protein constitutive coupling

The GPCR/G-protein interaction assessed via BRET in cell membranes is abolished by the addition of guanine nucleotides. We thus examined the effect of GDP on NOP/ G-protein interaction to investigate the extent of constitutive activity in the NOP receptor under the present experimental conditions. GDP up to 10 μM did not significantly modify the basal BRET ratio, and only a very weak inhibitory effect (5% of basal BRET) was detected by prolonging incubation time to 15 min.

### Effect of Pertussis-toxin treatment

**Fig 2:**
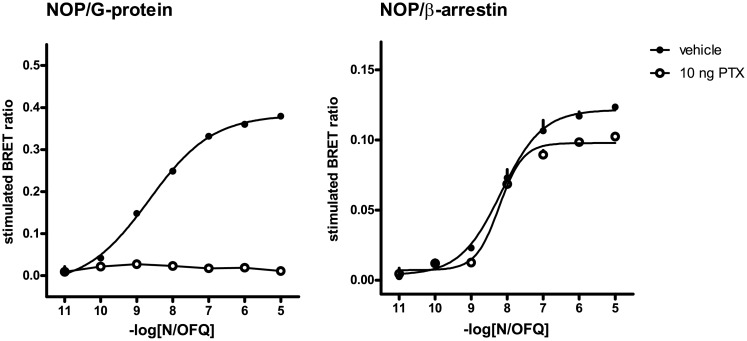
Concentration-response curves to N/OFQ—experiments performed in absence and in presence of PTX 10 ng 20 h treatment. NOP/G-protein (left panel) and NOP/β-arrestin 2 interaction experiments (right panel). Data are mean ± sem of 3 separate experiments performed in duplicate.

In order to elucidate the identity of the endogenous Gα subunits mediating the NOP/Gβ_1_ interaction, and to evaluate their potential effects on NOP mediated β-arrestin 2 recruitment, HEK293 cells stably expressing the human NOP (NOP/RLuc) receptor and either the Gβ_1_ subunit (Gβ_1_/RGFP) or the β-arrestin 2 (β-arrestin 2/RGFP) were treated for 20 h with 10 ng PTX. As shown in Fig 2, right panel, PTX treatment abolished the ability of the agonist to stimulate NOP/G-protein interaction, but had negligible effects on the stimulation of NOP/β-arrestin 2 interactions (Fig 2).

### Effects of different incubation times

In a preliminary series of experiments concentration response curves to N/OFQ, Ro 65–6570, and UFP-112 were performed at 5, 10, and 15 min of incubation and no major differences in agonist potency or efficacy were detected at both NOP/G-protein and NOP/β-arrestin 2 interaction. Thus further experiments were performed with a 5 min incubation time.

### Effect of ligands on NOP/G-protein interaction

**Fig 3:**
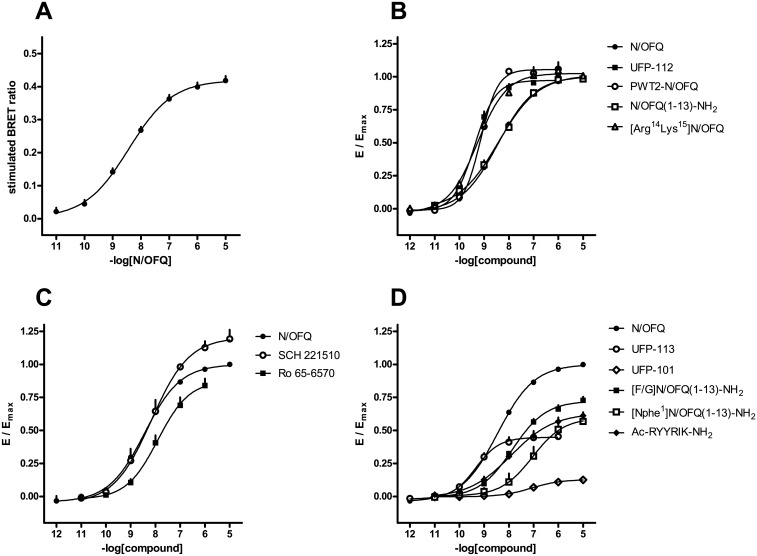
NOP receptor/G-protein interaction experiments—Concentration-response curves to N/OFQ (Panel A); N/OFQ, UFP-112, PWT2-N/OFQ, N/OFQ(1–13)-NH_2_, and [Arg^14^Lys^15^]N/OFQ (Panel B); N/OFQ, SCH-221510, and Ro 65–6570 (Panel C); N/OFQ, UFP-113, UFP-101, [F/G]N/OFQ(1–13)-NH_2_, [Nphe^1^]N/OFQ(1–13)-NH_2_, and Ac-RYYRIK-NH_2_ (Panel D). Data are expressed as mean ± sem of at least 5 separate experiments performed in duplicate.

Membrane extracts taken from HEK293 cells stably expressing both the human NOP (NOP/RLuc) receptor and the Gβ_1_ subunit (Gβ_1_/RGFP) were used to perform concentration-response curves to NOP ligands. The endogenous NOP receptor agonist N/OFQ promoted NOP/G-protein interaction in a concentration-dependent manner. N/OFQ displayed high potency (pEC_50_ 8.44) and a maximal effect which corresponded to a stimulation of 0.42 ± 0.01 BRET ratio units over the baseline (Fig 3A). Under the same experimental conditions synthetic peptides such as, [Arg^14^Lys^15^]N/OFQ, PWT2-N/OFQ, UFP-112, and N/OFQ(1–13)-NH_2_ produced similar stimulatory effects, with E_max_ values comparable to those induced by the natural peptide. N/OFQ(1–13)-NH_2_ was equipotent with N/OFQ, while [Arg^14^Lys^15^]N/OFQ, PWT2-N/OFQ, and UFP-112 were 7, 5, and 8 fold more potent than N/OFQ (Fig 3B). The non-peptide NOP ligands SCH-221510, Ro 65–6570, J-113397, SB-612111, C-24, and the opioid receptor antagonist naloxone were also investigated. SCH-221510 and Ro 65–6570 exhibited maximal effect not significantly different from those of N/OFQ, but were 2 and 5 fold less potent then the natural peptide (Fig 3C). In contrast, J-113397, SB-612111, C-24, and naloxone did not modify the basal BRET ratio. In a separate series of experiments the peptides UFP-101, UFP-113, [Nphe^1^]N/OFQ(1–13)-NH_2_, Ac-RYYRIK-NH_2_, and [F/G]N/OFQ(1–13)-NH_2_ were tested. All such peptides exhibited maximal effects that were significantly lower than that of N/OFQ, ranging from 0.14 (UFP-101) to 0.72 ([F/G]N/OFQ(1–13)-NH_2_). As far as potency is concerned, all these ligands were less potent than the natural peptide, with the exception of UFP-113, which was 8 fold more potent than N/OFQ (Fig 3D). However, due to its very low E_max_ value, the potency of UFP-101 could not be precisely estimated. All the data obtained in this series of experiments have been summarized in Table 2.

**Table 2:** Potencies (pEC_50_), concentration ratio (CR), and maximal effects (E_max_) of the compounds tested on the interaction of NOP with G protein and β-arrestin 2.

	G protein			β-arrestin 2		
	pEC_50_ (CL_95%_)	CR	E_max_ ± sem	pEC_50_ (CL_95%_)	CR	E_max_ ± sem
N/OFQ	8.44 (8.33–8.56)	1	1	8.02 (7.81–8.23)	1	1
N/OFQ(1–13)-NH_2_	8.46 (7.94–8.99)	0.95	1.00 ±0.03	8.02 (7.72–8.32)	1	1.00 ±0.08
[Arg^14^Lys^15^]N/OFQ	9.27 (9.21–9.33)	0.15	1.00 ±0.05	7.83 (7.63–8.03)	1.55	1.10 ±0.04
UFP-112	9.35 (9.10–9.60)	0.12	0.98 ±0.03	8.37 (8.18–8.57)	0.45	0.89 ±0.07
PWT2-N/OFQ	9.17 (8.97–9.48)	0.19	1.10 ±0.01	7.53 (7.30–7.77)	3.09	1.3 ±0.07
[F/G]N/OFQ(1–13)-NH_2_	7.85 (7.75–7.96)	3.89	0.72* ±0.03	inactive		
UFP-113	9.35 (9.29–9.41)	0.12	0.45* ±0.04	inactive		
Ac-RYYRIK-NH_2_	7.90 (7.45–8.34)	3.39	0.63* ±0.02	inactive		
[Nphe^1^]N/OFQ(1–13)-NH_2_	6.85 (6.70–6.99)	38.9	0.55* ±0.04	inactive		
UFP-101	7.01 (6.79–7.24)	26.9	0.14* ±0.04	inactive		
Ro 65–6570	7.77 (7.35–8.18)	4.68	0.96 ±0.05	6.37 (6.08–6.65)	44.67	0.84 ±0.06
SCH-221510	8.26 (7.06–9.46)	1.51	1.20±0.03	6.96 (6.43–7.48)	11.48	0.75 ±0.10
J-113397	inactive			inactive		
SB-612111	inactive			inactive		
C-24	inactive			inactive		
naloxone	inactive			inactive		

Inactive means the compound did not stimulate BRET ratios up to 10 μM.

*p < 0.05 vs. N/OFQ, according to one way ANOVA followed by the Dunnett's test for multiple comparison.

Ligands with very weak or no agonist effects were assessed as antagonists.

In a series of pilot experiments, UFP-101, SB-612111 and C-24 were tested at fixed concentrations against the concentration-response curve of N/OFQ. All ligands produced the expected shift of agonist EC_50_, however, unlike UFP-101, SB-612111 and C-24 also caused a depression of the maximal effect elicited by N/OFQ under such conditions. On repeating the experiments by increasing the time of incubation from 5 to 15 min, the decrease of N/OFQ E_max_ value was no longer evident. Thus, a longer incubation time was used to assess antagonist potency for both G protein interaction and arrestin interaction (see below).

**Fig 4:**
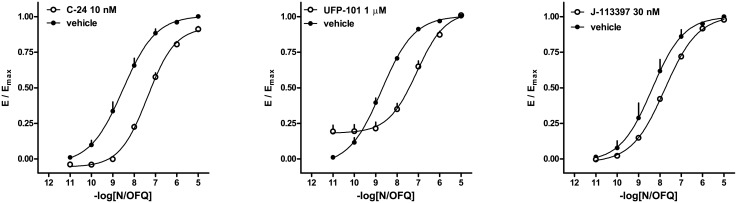
NOP receptor/G protein interaction experiments—concentration-response curves to N/OFQ in absence and in presence of C-24, UFP-101, and J-113397. Data are mean ± sem of 5 separate experiments performed in duplicate.

The ligands C-24 (10 nM), UFP-101 (1 μM), and J-113397 (30 nM) added to the concentration-response curve of N/OFQ produced a rightward shift of the curve without significantly changing the maximal effect. From these experiments the following pK_B_ values were derived: C-24, 9.11, UFP-101, 7.66, and J-113397, 7.95 (Fig 4). In similar experiments naloxone (1 μM) did not modify the concentration response curve to N/OFQ.

**Fig 5:**
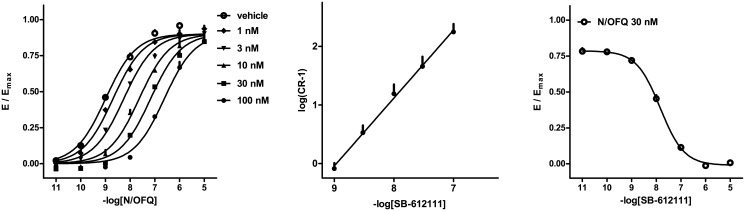
NOP receptor / G protein interaction experiments—concentration-response curves to N/OFQ in absence and in presence of increasing concentrations (1–100 nM) of SB-612111 (left panel). The corresponding Schild plot is shown in the middle panel. The inhibition response curve to SB-612111 vs. N/OFQ 30 nM is shown in the right panel. Data are mean ± sem of 4 separate experiments performed in duplicate.

Schild analysis was used to obtain a more detailed analysis of the antagonist properties of SB-612111. Concentration-response curves of N/OFQ were generated in the absence and presence of increasing concentrations (1–100 nM) of the antagonist. SB-612111 produced a parallel rightward shift of the N/OFQ curves in a concentration dependent manner, without modifying the maximal effect elicited by the agonist (Fig 5, left panel). The resulting Schild plot was linear with a slope value of 1.16 ± 0.03; a pA_2_ value of 8.96 was derived from these experiments (Fig 5, middle panel). Finally, the antagonist potency of SB-612111 was also estimated from inhibition response curves. Increasing concentrations of SB-612111 (10 pM—10 μM) were tested against a fixed concentration of N/OFQ (30 nM); the pK_B_ derived from these experiments was 9.13 (Fig 5, right panel). All the data obtained in this series of experiments are summarized in Table 3.

**Table 3:** Effects on NOP/G protein and NOP/β-arrestin 2 interactions of NOP ligands showing reduced efficacy.

	G protein			β-arrestin 2		
	pEC_50_	E_max_	pK_B_ (CL_95%_)	pEC_50_	E_max_	pK_B_ (CL_95%_)
N/OFQ	8.44	1.00	**-**	8.02	1.00	**-**
[F/G]N/OFQ(1–13)-NH_2_	7.85	0.72*	**-**	inactive		7.52 (6.88–8.16)
UFP-113	9.35	0.45*	**-**	inactive		9.42 (8.44–10.40)
Ac-RYYRIK-NH_2_	7.90	0.63*	**-**	inactive		7.11 (6.85–7.38)
[Nphe^1^]N/OFQ(1–13)-NH_2_	6.85	0.55*	**-**	inactive		7.00 (6.33–7.66)
UFP-101	7.01	0.14*	7.66 (7.23–8.10)	inactive		7.34 (6.73–7.94)
J-113397	inactive		7.95 (6.06–9.83)	inactive		7.27 (6.24–8.30)
SB-612111	inactive		8.96 (8.84–9.08)	inactive		7.91 (7.26–8.56)
C-24	inactive		9.11 (8.19–10.05)	inactive		9.09 (8.56–9.63)
naloxone	inactive		inactive	inactive		inactive

Inactive means that the compound was inactive up to 1 μM.

*p < 0.05 according to one-way ANOVA followed by the Dunnett test.

### Comparison of the pharmacological profile of ligands with previous data

The pharmacological activity of ligands for promoting NOP receptor-G protein interaction was found to be in close agreement with the available data obtained in the [^35^S]GTPγS binding assay. In fact the potency of N/OFQ in evoking stimulation of BRET ratio (pEC_50_ 8.44) is very similar to that previously reported for the stimulation of [^35^S]GTPγS binding (e.g. 8.95, [46]). Moreover, compounds such as UFP-112, [Arg^14^Lys^15^]N/OFQ, PWT2-N/OFQ, and N/OFQ(1–13)-NH_2_ behaved as full agonists in both assays and in both exhibited an identical rank order of potency: UFP-112 > [Arg^14^Lys^15^]N/OFQ > PWT2-N/OFQ > N/OFQ(1–13)-NH_2_ = N/OFQ [8,26]. Likewise, the results observed for number of synthetic peptides, known as NOP partial agonists, were also in best agreement with pharmacological activities characterized in [^35^S]GTPγS binding assays. The order of potency measured for these ligands (UFP-113 > Ac-RYYRIK-NH_2_ > [F/G]N/OFQ(1–13)-NH_2_ > [Nphe^1^]N/OFQ(1–13)-NH_2_) is in line with previously reported results [8]. The very high potency exhibited by UFP-113 in the BRET assay (pEC_50_ 9.35) associated with a relative efficacy of 0.45, is also consistent with the similar profile reported in [^35^S]GTPγS binding experiments (pEC_50_ 9.73, E_max_ 0.79, [30]. Non-peptide ligands such as Ro 65–6570 and SCH-221510 displayed similar maximal effects but a reduced potency (5 and 2 fold lower, respectively) compared to N/OFQ. The same results were observed previously using a [^35^S]GTPγS binding assay [28].

The correspondence between BRET and GTPγS activities is also evident on comparing the estimates of antagonist potencies (pK_B_ values). For example, the BRET assay potency of SB-612111 estimated either using a classic Schild protocol (pA_2_ 8.96) and from inhibition-response curves (pK_B_ 9.13) was compatible with the previous estimate (9.70) obtained in [^35^S]GTPγS binding studies [47].

### Effect of ligands on NOP/β-arrestin 2 interactions

**Fig 6:**
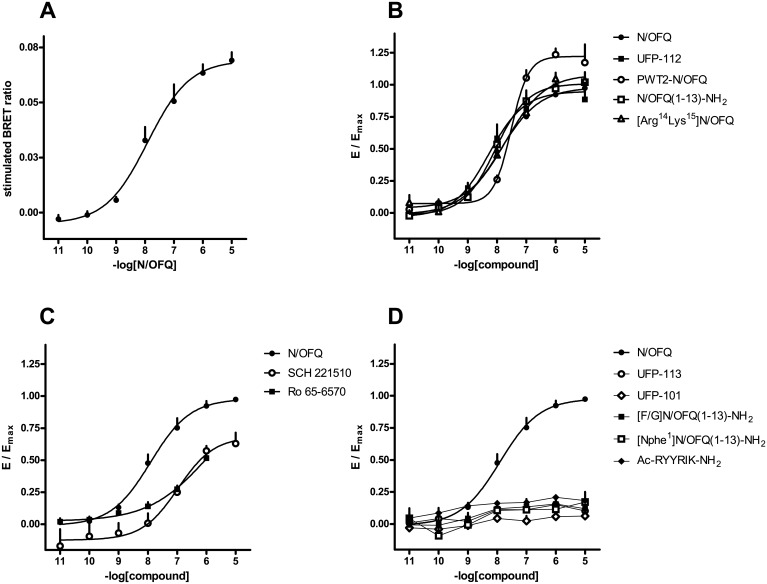
NOP receptor/β-arrestin 2 interaction experiments—Concentration-response curves to N/OFQ (Panel A); N/OFQ, UFP-112, PWT2-N/OFQ, N/OFQ(1–13)-NH_2_, [Arg^14^Lys^15^]N/OFQ (Panel B); N/OFQ, SCH-221510, and Ro 65–6570 (Panel C); N/OFQ, UFP-113, UFP-101, [F/G]N/OFQ(1–13)-NH_2_, [Nphe^1^]N/OFQ(1–13)-NH_2_, and Ac-RYYRIK-NH_2_ (Panel D). Data are expressed as mean ± sem of at least 5 separate experiments performed in duplicate.

**Fig 7:**
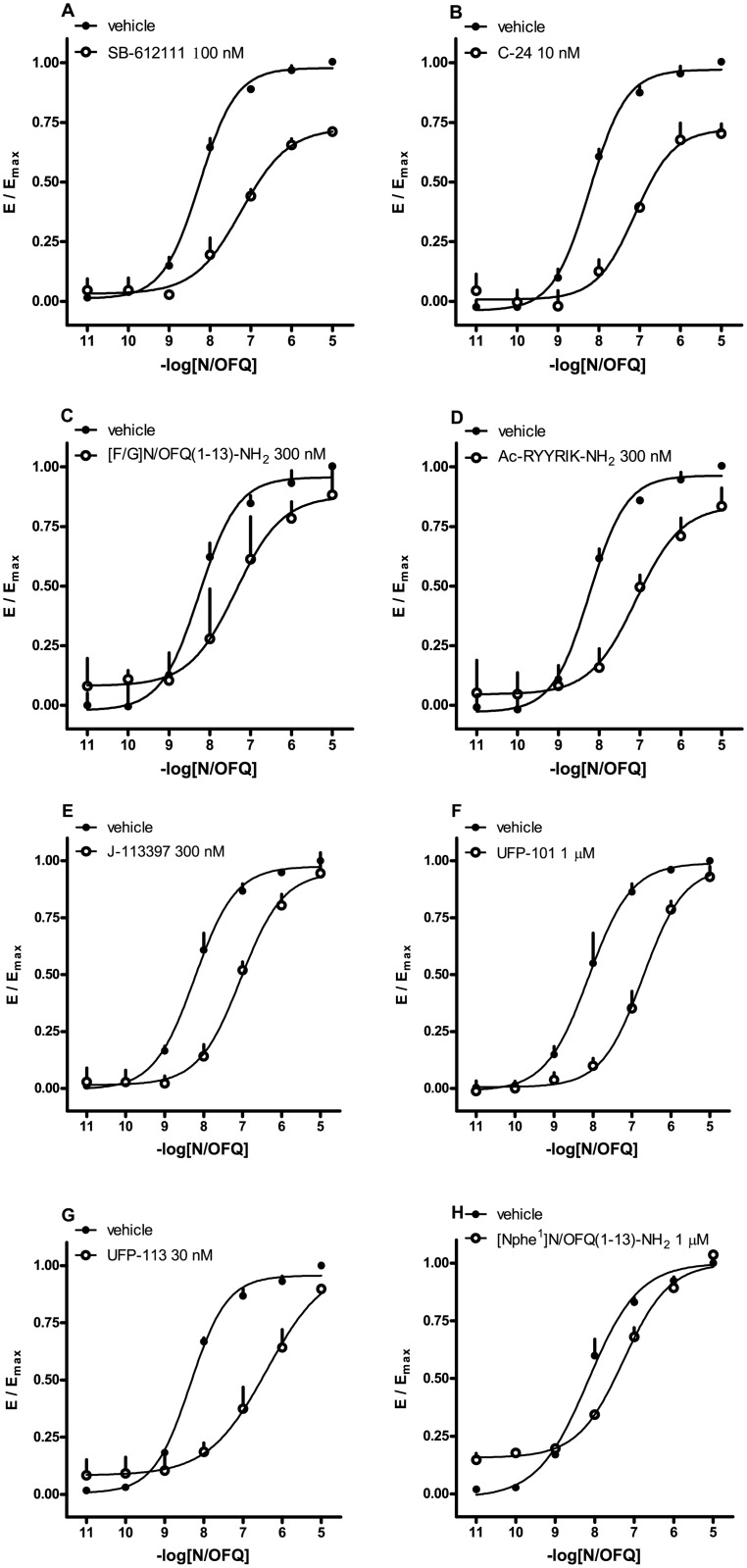
NOP receptor / β-arrestin 2 protein interaction experiments—concentration-response curves to N/OFQ in absence and in presence of SB-612111 (100 nM, panel A), C-24 (10 nM, panel B), [F/G]N/OFQ(1–13)-NH_2_ (300 nM, panel C), Ac-RYYRIK-NH_2_ (300 nM, panel D), J-113397 (300 nM, panel E), UFP-101 (1 μM, panel F), UFP-113 (30 nM, panel G), and [Nphe^1^]N/OFQ(1–13)-NH_2_ (1 μM, panel H). Data are mean ± sem of at least 3 separate experiments performed in duplicate.

HEK293 cells stably expressing both the human NOP (NOP/RLuc) receptor and the β-arrestin 2 (β-arrestin 2/RGFP) protein were used for performing concentration-response curves to NOP ligands. N/OFQ promoted receptor/arrestin interaction in a concentration dependent manner displaying high potency (pEC_50_ 8.02), and maximal effects that corresponded to a stimulation of 0.07 ± 0.004 BRET ratio units over the baseline (Fig 6A). The synthetic peptides [Arg^14^Lys^15^]N/OFQ, UFP-112, and N/OFQ(1–13)-NH_2_ mimicked the stimulatory effect of N/OFQ and showed similar maximal effects. With regards to potency, UFP-112 was slightly more potent, whereas [Arg^14^Lys^15^]N/OFQ and PWT2-N/OFQ were 2 and 3 fold less potent than N/OFQ (Fig 6B). The non-peptide compounds SCH-221510, Ro 65–6570, J-113397, SB-612111, C-24, and naloxone were also investigated for their ability to promote NOP receptor/β-arrestin 2 interactions. SCH-221510 was 10 fold less potent than N/OFQ (Fig 6C) and Ro 65–6570 exhibited a similar decrease of potency, although we should note that the incomplete concentration-response curves of this compound did not allow an experimentally verified assessment of the asymptotic plateau, thus the pEC_50_ and E_max_ values obtained for this compound (6.3 and 0.84) are extrapolated from the fitting routine. J-113397, SB-612111, C-24, and naloxone did not modify the basal BRET ratio. The synthetic peptides that are partial agonist at G protein coupling (i.e., [F/G]N/OFQ(1–13)-NH_2_, [Nphe^1^]N/OFQ(1–13)-NH_2_, UFP-101, UFP-113, [Nphe^1^]N/OFQ(1–13)-NH_2_, and Ac-RYYRIK-NH_2_) showed only a variable and weak stimulation of receptor / arrestin interaction (Fig 6D). All such ligands were thus analyzed as antagonists. They behaved as competitive antagonists, by producing a rightward shift in the concentration-response curve of N/OFQ for arrestin coupling without affecting the E_max_ value. The following pA_2_ values were computed from such experiments: [F/G]N/OFQ(1–13)-NH_2_, 7.52, Ac-RYYRIK-NH_2_, 7.11, [Nphe1]N/OFQ(1–13)-NH_2_, 7.00, UFP-101, 9.42 (Fig 7). All the data obtained in this series of experiments have been summarized in Table 2. Of note, the antagonist potencies estimated for these ligands in inhibiting arrestin coupling are very close to their agonistic potency in stimulating G protein coupling. This indicates that the lack of agonistic effect on arrestin is not due an insufficient concentration of ligand that was used in the assay.

We also measured the inhibitory potency for arrestin coupling of the other antagonists, such as J-113397, SB-612111, C-24, J-113397, and, naloxone. With the exception of the latter, which was inactive, all antagonists produced the expected competitive inhibition with a rightward shift of the concentration-response curve of N/OFQ, from which pA_2_ values were computed. All the data obtained in this series of experiments are summarized in Table 3.

## Discussion

In the present study the pharmacological profile of the human NOP receptor was investigated using a BRET assay, which is based on the fusion of a RLuc donor to the receptor and a RGFP acceptor to the transduction protein. The same technology was employed to investigate the interaction of the NOP receptor with Gβ_1_ subunits (which is mediated by endogenous Gα subunits of the membrane) and with β-arrestin 2 (which is recruited to the membrane upon receptor activation). Thus, in order to exclude cross interaction between transduction proteins, receptor/G-protein interactions were studied in isolated membranes while receptor/arrestin interactions were determined in whole cells. The pharmacological profile for NOP receptor coupling to the two transduction proteins was evaluated using a large panel of peptide and non-peptide ligands, all of which are selective for the NOP receptor and enclose molecules that are known to display a broad range of receptor efficacy, from full agonism to inverse agonism. This allows an exhaustive and meaningful comparison of the molecular characterization of NOP receptor activity presented in this study with pharmacological results reported in a variety of previous investigations.

### Methodological aspects

Control experiments indicate that the BRET signals reported here represent an accurate determination of the ligand-induced changes of NOP receptor coupling to the two fluorescently-tagged transduction proteins, and that the comparison of such activities are not biased by large differences in receptor expression between the cell clones employed in this work. In fact, we found that within the range of concentrations used in this study ligands did not exert unspecific effects on the enzymatic activity of the Rluc used as BRET donor [48]. Moreover, the analysis of the intrinsic luminescence of the cell membranes, which quantifies the abundance of Rluc-tagged receptor, indicated that the NOP receptors were expressed at comparable levels in the two cell clones.

The NOP/Gβ_1_ BRET signal measured in cell membrane preparations cannot be influenced by β-arrestin 2, since the amount of this transducer in the plasma membrane is negligible prior to ligand activation of the receptor. However, the NOP/Gβ_1_ interaction may be mediated by a plurality of Gα subunits that can interact with the receptor. As shown here, PTX treatment abolished the BRET signal, indicating that the measured activity is primarily accounted for by the pertussis-sensitive family of Gα subunits (i.e., Gα_i/o_). Thus the pharmacological parameters of NOP/G protein interaction reported here can be meaningfully compared with the results of pertussis toxin-dependent signalling studies of the NOP receptor [49–51]. In contrast, the NOP/β-arrestin 2 signal measured in whole cells was resistant to the toxin, indicating that the interaction with G protein does not interfere with the measurement or receptor-arrestin interactions. In conclusion, unlike the results of studies based on measurements of signalling activities occurring downstream of the transduction proteins, the receptor-transducer coupling activities reported in this study represent determinations that are largely unaffected by the mutual antagonism existing between arrestin and G protein for complex formation with the receptor. Thus, these results are independent estimates of the ability of NOP receptor to associate with each of the two proteins.

### NOP / G-protein interaction

In the present study we found that GDP was not able to significantly inhibit the baseline of BRET ratio in membranes from cells expressing NOP-RLuc and Gβ_1_-RGFP. This indicates that the level of spontaneous coupling between the NOP receptor and G proteins is negligible in isolated membranes, and stands in contrast with data reported earlier on delta/RLuc or mu/RLuc opioid receptors using the same type of BRET assay [52]. This result suggests that the NOP receptor has very low propensity to adopt a constitutively active conformation, particularly if compared to the delta receptor. Also in support of this suggestion is the observation that the ligand C-24, which was previously reported to behave as NOP inverse agonist [53], did not produce any significant inhibition of the basal BRET ratio. In agreement with such a conclusion, little evidence for NOP receptor constitutive activity has been described so far, at least under physiological conditions. Data suggesting NOP constitutive activity were only obtained by electrophysiological recording of neurons in which the over expression of the receptor was induced by microinjection of coding cDNA [53]. In another study, in which the ability to constitutively activate G-protein-coupled pathways was investigated in a series of NOP receptor point mutations, only the N133W mutant displayed increased ligand-independent signalling [54]. Interestingly, this mutated residue (N3.35) was recently found to contribute to the network of interactions that establish a sodium binding pocket in the structure of several GPCRs [55], including the delta opioid receptor [56]. It was suggested that sodium binding may favor the inactive conformation of the receptor [55]. Thus, additional comparative BRET experiments on the G protein interactions of delta and NOP receptors bearing cross-mutations in the residues that generate the sodium pocket will be necessary to evaluate if this structural domain is responsible for the large difference in constitutive activity observed between the two receptor subtypes.

In the first part of this study we appraised the pharmacological profile of the ligands in promoting NOP receptor interaction with the pertussis toxin-sensitive family of G-protein. As detailed in the Results section, the pharmacological parameters derived from this investigation are in best agreement with the results previously reported from the analysis of GTPγS binding data [8,26,46]. This finding is not surprising, as both assays are performed in isolated membranes and both measure the same early event of the signalling cascade, i.e., receptor-mediated G-protein activation. Yet, the good correlation that we found in this study is important, because it suggests that there is no dissociation between ligand-induced coupling to the G protein and ligand-promoted changes in the nucleotide-exchange properties of the G protein, within the studied ligands. In other words, we found no evidence for the existence of ligands that on promoting receptor-G protein coupling might produce a non proportional or even opposite effect on G protein activation. Moreover, this satisfactory agreement between NOP/G protein coupling and NOP stimulated GTPγS binding further demonstrate that the BRET assay used in the study provides a robust and precise assessment of the ligand ability to activate the NOP receptor.

On comparing the data with a wider range of pharmacological assays carried in vitro or in vivo, noticeable discrepancies are only apparent for a number of synthetic peptides that are known as NOP partial agonists. Such divergences are likely explained by the variations in stimulus/response coupling efficiency that characterize different pharmacological preparations. In fact, in accordance with classical receptor theory, the effect of a partial agonist can range from none to an almost full response, depending on the extent of amplification that the sensitivity of the signaling pathway exerts on the initial biological signal triggered by the receptor in complex with the transduction protein. For example, the peptide analog [F/G]N/OFQ(1–13)-NH_2_ that shows a significant level of partial agonism (E_max_ 0.72) in this study, was previously reported to behave as pure antagonist in a low-efficiency coupled preparation, such as the electrically stimulated mouse vas deferens [29]. The same ligand displayed varying level of efficacy in other studies in vitro or in vivo [8]; it was also specifically shown that the relative response of this agonist can be varied on manipulating the levels of NOP receptor expression, which is a typical feature of partial agonism [51]. Analogous considerations apply to UFP-113, which shows weak and variable agonism in the mouse vas deferens [30], or to the hexapeptide Ac-RYYRIK-NH_2_, for which conflicting and variable levels of efficacy were reported in the literature [31,57]. Also consistent with this interpretation are the results obtained with the analog UFP-101. Although this peptide is known to behave as pure antagonist in a vast range of pharmacological tests [8,58], it displayed a faint but detectable level of residual efficacy (E_max_ 0.14) in our assay. This is in line with the observation that a weak partial agonism in UFP-101 could only be revealed after receptor over-expression in neurons microinjected with plasmid coding for the NOP sequence [53].

In conclusion, we suggest that the rank order of relative effects that we measured for these partial agonists in the BRET assay (i.e. [F/G]N/OFQ(1–13)NH_2_ > Ac-RYYRIK-NH_2_ > [Nphe^1^]N/OFQ(1–13)-NH_2_ > UFP-113 >> UFP-101, see table 2) provides a more accurate description of the level of efficacy endowed in these ligands.

Also the estimates of antagonist potency derived from BRET analysis were in substantial agreement with similar determinations made in a variety of different pharmacological studies. However, one interesting observation is that two of such antagonists, SB-612111 and C-24, displayed a pattern of agonist inhibition suggesting insurmountable behavior under some assay conditions (i.e. 5 min incubation with agonist); this is in contrast with a plurality of previous studies, were the competitive nature of these compounds was demonstrated [35,46,47]. We found, however, that a 3-fold increase in incubation time was sufficient to restore an essentially competitive pattern of antagonism in these compounds, while other antagonists with lower potency, such as UFP-101 or J-113397, did not show similar time-dependent changes. Thus, it is conceivable that the very slow dissociation rate of these ligands and the consequent effect on the agonist ability to reach a steady-state level of receptor occupancy are responsible for the phenomenon. Also in line with these findings is the intriguing observation that the antagonist action of UFP-101 in the isolated mouse vas deferens is immediately reversible on washing, while the effect of SB-612111 remains unchanged even after 3 hours of repeated washing [47].

### NOP / β-arrestin 2 interaction

In the second part of the study, we report the pharmacological profile of the same panel of ligands for the induction of NOP receptor interaction with β-arrestin 2. Although this is the first study in which a systematic assessment of the efficacy of NOP agonists for arrestin was made, previous results based on NOP receptor internalization show that agonists active in promoting internalization of the NOP receptor [59,60] are among those that in this study display a robust effect on arrestin coupling. This supports the notion that NOP receptor internalization requires a clathrin-dependent rapid endocytosis mechanism that is mediated by arrestins [61].

The most prominent finding in our data is that the agonists showing relative E_max_ values ≤ 0.7 in G protein coupling produced negligible or no effects on arrestin recruiting. In fact, we also found that these inactive ligands behaved as virtually pure competitive antagonists in inhibiting the effect of the endogenous agonist N/OFQ on arrestin interaction. These results remind similar observations reported for delta and mu receptor interaction with arrestin, where the relationships between G protein and arrestin couplings for both receptors were strongly hyperbolic [22]. Perhaps this hyperbolic relation may be stronger in the NOP receptor, judging from the greater threshold of partial G protein agonism that is necessary in order to observe a measurable effect on arrestin interaction. In addition, our results indicate that the potencies of agonists for NOP/arrestin interaction were systematically lower that those measured for NOP/G protein interaction, by factors ranging between 0.4–1.6 log units.

As far as pure antagonists are concerned, these compounds were similarly active at NOP/G protein and NOP/arrestin interaction and displayed an identical rank order of potency i.e. C-24 > SB-612111 > J-113397 ≥ UFP-101, with potency values that were only slightly lower at NOP/β-arrestin 2 than NOP/G-protein.

Altogether the comparison of the pharmacological profile of NOP receptor interacting with G-protein or with β-arrestin 2 suggests minor differences for receptor antagonists, loss of efficacy for partial agonists and decrease of potency for synthetic full agonists. Collectively, these findings indicate that the activation induced by most NOP agonists is significantly biased towards promoting receptor-G protein interaction rather than receptor-arrestin interaction. Thus, the question is whether this preference is caused by system bias or reflects a true difference in agonist efficacy for driving the NOP receptor to interact with the two transduction proteins.

Two common sources of system bias are the difference in the efficiency of the signalling pathway that couples the activation of each transducer to the measured biological signals, or the difference in the sensitivity of the assay methods employed to evaluate divergent transduction pathways. Yet, neither kind of system bias is likely to affect the results presented in this study. In fact, the BRET signals reported here quantify the extent of protein-protein interaction between receptor and transducer; this is not influenced, unlike biological responses, by the differences in efficiency of the signalling pathways. Furthermore, the methodology adopted in this study is based on exactly the same pair of donor/acceptor reporters that were used to both assess G protein and arrestin interactions. Thus, it is unlikely that major differences in the efficiency of the resonance energy transfer system may alter the sensitivity of the two determinations.

However, there is a third source of system bias that may play a fundamental role in our results: i.e., the difference in binding affinity for the interaction of the two transduction proteins with the empty receptor. On comparing G protein vs. arrestin interactions, this difference is particularly difficult to evaluate, because under the generic term “affinity” one must factor additional biochemical events that regulate the ability of arrestin to associate to the receptor, such as GRK-mediated phosphorylation and the intracellular process of translocation that brings arrestin to the receptor in the plasma membrane. Thus, it is possible that a globally lower susceptibility of the NOP receptor to be docked by arrestin than by G protein might generate the systematic reduction in agonist effects observed here, despite a conserved efficacy of the ligands in promoting the two interactions.

A qualitative criterion that helps to distinguish between ligand and system bias is the rank of ligand effects at the two transduction systems; in fact, system bias, regardless of the source, cannot alter this ordering. An inversion in the rank order of potency was noted for some agonists. For example, [Arg^14^Lys^15^]N/OFQ and PWT2-N/OFQ were more potent than the natural agonist in promoting G protein interaction, but less potent than N/OFQ in inducing arrestin interaction. In contrast, UFP-112 and the non peptide agonists (SCH-221510 and Ro-656570) maintained the same rank of potency at the two transduction proteins.

### Biased agonism

To obtain a quantitative estimate of biased efficacy, approximate values for the difference in intrinsic efficacies of the agonists for the two transduction proteins (i.e. ε_Gprotein_/ε_arrestin_) were computed as bias factors. Agonists such as Ro-656570 and PWT2-N/OFQ, displayed a 10-fold greater efficacy for G protein interaction. Smaller differences were observed in other agonists, such as UFP-112 and SCH-221510, whereas no biased efficacy was found in N/OFQ(1–13)-NH_2_ (Table 4). Although the large propagated error in this calculation prevents an accurate assessment of the significance of the computed differences, the data suggest that several synthetic agonists, including perhaps those that behave as pure antagonists of receptor-arrestin interaction, may display significant losses of intrinsic efficacy at this transduction protein. This trend might result from the fact that these synthetic NOP analogs were designed and selected on the basis of SAR studies derived from G-protein-dependent signalling assays. Thus, it is possible that SAR studies focused on receptor/β-arrestin interaction will allow the discovery of arrestin biased agonist for the NOP receptor in the future

**Table 4:** Bias factors obtained from at least 5 independent E_max_/EC_50_ values from both NOP/G-protein and NOP/arrestin experiments.

	bias factor ± sem
N/OFQ	0.00
N/OFQ(1–13)-NH_2_	0.00 ± 0.40
[Arg^14^,Lys^15^]N/OFQ	0.25 ± 0.46
UFP-112	0.71 ± 0.37*
SCH 221510	0.77 ± 0.75
Ro-65 6570	1.07 ± 0.38*
PWT2-N/OFQ	1.09 ± 0.28*

*p < 0.05 according to the Student t test for paired data.

## Conclusions

Ligand bias has important implications in drug development. In principle, using biased agonists that selectively activate a single transduction pathway it might be possible to maximize therapeutically useful responses and minimize side effects. Some examples of this innovative strategy were already described in literature. G-protein biased agonists acting at the mu opioid receptor are under development as analgesics with higher tolerability [19] while G-protein biased agonists at GPR109 may reduce serum fatty acids without inducing cutaneous flushing [62]. Similarly, β-arrestin biased agonists acting at the AT1 receptor may be effective drugs for the treatment of heart failure [10], while biased β-arrestin PTH receptor ligands are potential innovative drugs for promoting bone formation [63]. N/OFQ via selective NOP receptor activation can control several biological functions, however the relative role of G-protein and arrestin in mediating these actions is presently unknown. Further studies are needed to identify new lead molecules that will help to understand the structural requirements underlying the difference in efficacy of NOP agonists for G-proteins and arrestins, and the potential therapeutic indications of G-protein or arrestin biased NOP agonists. N/OFQ can produce robust antinociceptive effects following spinal administration both in rodents and non human primates [64]. Thus, it is important to clarify whether the extent of G-protein bias, which is present in some NOP agonists as shown in this study, may be a crucial determinant for the antinociceptive response; this may lead to the discovery of innovative spinal analgesics. Interestingly, compounds such as UFP-112 and PWT2-N/OFQ demonstrated robust and extremely long acting antinociceptive properties after spinal administration in rodents and monkeys [24,26,65]. It has been suggested that the long lasting action of these compounds may reflect reduced susceptibility to peptidase action [24,26]. However, according to the present data, we may speculate that the G protein bias nature of these compounds could contribute to their persistent antinociceptive effect. Further studies and the use of mice knockout for the β-arrestin 2 gene are needed to validate this hypothesis.
